# Investigating a hyper-endemic focus of *Taenia solium* in northern Lao PDR

**DOI:** 10.1186/1756-3305-7-134

**Published:** 2014-03-28

**Authors:** Anna Okello, Amanda Ash, Chattouphone Keokhamphet, Emma Hobbs, Boualam Khamlome, Pierre Dorny, Lian Thomas, John Allen

**Affiliations:** 1 CSIRO Animal, Food and Health Sciences, Australian Animal Health Laboratory (AAHL) Regional Programme, 5 Portarlington Road, East Geelong, Victoria, Australia; 2 National Animal Health Laboratory, Department of Livestock and Fisheries, Lao PDR, Vientiane, Lao; 3 School of Veterinary and Life Sciences, Murdoch University, Perth, Australia; 4 School of Biomedical Sciences, College of Medicine and Veterinary Medicine, University of Edinburgh, Chancellors Building 49 Little France Crescent, Edinburgh EH16 4SB, Scotland; 5 Department of Hygiene and Prevention, Ministry of Health, Lao PDR, Vientiane, Lao; 6 Veterinary Helminthology Unit, Department of Biomedical Sciences, Institute of Tropical Medicine, Nationalestraat 155, 2000 Antwerp, Belgium; 7 International Livestock Research Institute, PO Box 30709, Nairobi 00100, Kenya; 8 Centre for Immunity, Infection and Evolution, University of Edinburgh, Kings Buildings, West Mains Rd, Edinburgh, Scotland EH9 3YJ

**Keywords:** *Taenia solium*, Taeniasis, Cysticercosis, Lao PDR, Neglected tropical diseases, One health

## Abstract

**Background:**

The *Taenia solium* cysticercosis-taeniasis complex is a Neglected Tropical Disease of significant public health importance in many impoverished communities worldwide. The parasite is suspected to be endemic in Lao PDR as a result of widespread risk factors including open human defecation, free ranging pigs and weak systems for meat inspection and carcass condemnation. Reported prevalences of human taeniasis throughout the country have ranged from 0-14%, although few of these have definitively diagnosed *T. solium,* grossly indistinguishable from *Taenia saginata* (beef tapeworm) and *Taenia asiatica.* This short communication details the suspicion of a hyper endemic “hotspot” of *T. solium* in a remote Tai Dam village in northern Lao PDR.

**Findings:**

Initial antibody serosurveillance of four provinces in Lao PDR in 2011 indicated human taeniasis and cysticercosis prevalences of 46.7% and 66.7% respectively, in the village of Om Phalong in the north of the country. Subsequent copro-antigen ELISA on 92 human faecal samples from this same village, representing a total 27.9% of the target community, indicated a taeniasis prevalence of 26.1% (95% CI?=?18.2-35.9). Subsequent PCR and sequencing of samples (n?=?5) all identified as *T. solium;* the other human tapeworms *T. saginata* and *T. asiatica* were not detected in any of the samples genotyped.

**Conclusion:**

This is potentially one of the highest documented prevalences of *T. solium* taeniasis to date in Lao PDR, if not the Southeast Asia region. This result raises suspicion that other “hotspots” of *T. solium* hyper endemicity may exist in the region, particularly in communities where the consumption of raw pork is commonplace as a result of cultural practices.

## Findings

### Background

The human taeniasis/cysticercosis complex, caused by the tapeworm parasite *Taenia solium,* is listed by the World Health Organisation (WHO) as one of eight Neglected Zoonotic Diseases (NZDs) of significant public health importance, for which a combined ‘One Health’ approach by the human and animal health sectors is recommended for control in endemic countries [1,2]. Humans can act as both the definitive and accidental dead-end intermediate hosts of the parasite, with a risk of infection from both the adult (taeniasis) and larval stages (cysticercosis), respectively. Poor sanitation, open defecation and free-ranging pig production systems contribute to the ingestion of tapeworm eggs by the human and pig intermediate hosts, leading to the development of larval cysts in both. In humans, a common site of cyst development is the brain, manifested as a condition known as neurocysticercosis (NCC) and deemed the most frequent preventable cause of acquired epilepsy in endemic countries [3,4]. In addition, ingestion of cystic pork through the consumption of raw or undercooked meat propagates parasite survival in at-risk human populations through increasing the population of taeniasis positive carriers. Advocacy and political commitment for the control of *T. solium* in endemic countries under various Neglected Tropical Disease (NTD) frameworks is growing, culminating with the World Health Assembly’s adoption of Resolution WHA66.12 on NTDs in May 2013. Prior to this, specific Action Points in the 2012 WHO NTD Roadmap referred to the requirement by 2015 for a “validated strategy for the control and elimination of *T. solium”*, with scaling up of interventions in selected countries by 2020 [5].

The Lao People’s Democratic Republic (Lao PDR) is a Southeast Asian country that relies heavily on agricultural production, with up to 70% of households in the northern part of the country involved in smallholder pig production [6]. The conditions perpetuating *T. solium* transmission are widespread, including free ranging pig production systems, poor sanitation and lack of meat inspection, particularly at informal markets. A number of surveys on human intestinal helminth infections in different parts of the country over the last 25 years have identified non-specific *Taenia* species, with prevalences ranging from 0 to 14% [6]. A more recent study across four northern provinces in Lao PDR found a human cysticercosis prevalence of 2.2% (95% CI?=?1.4-3.0) and taeniasis prevalence of 8.4% (95% CI?=?6.9-9.9) [7].

In 2011, a large scale multi-disease serosurvey of pigs and humans was undertaken in Savanakhet and Luang Prabang provinces of Lao PDR, with additional samples from eight villages in Phongsaly and Xayaboury provinces in the far north and west, under collaboration between the Australian Centre for International Agricultural Research (ACIAR) and the International Livestock Research Institute (ILRI) [8]. Over 1000 human serum samples (n?=?1012) from the four provinces were tested for a range of pig zoonoses, including the use of an Enzyme-linked Immunoelectrotransfer Blot strip (EITB, US Centers for Disease Control, Atlanta, Georgia) to determine previous exposure of human participants to *T. solium.* EITB is reported to be highly specific (100%) and sensitive (97%) to the detection of circulating *T. solium* taeniasis antibodies, with a cysticercosis specificity and sensitivity of 99.4% and 97% respectively, provided at least two cysts are in the brain [9]. A number of hotspots were identified amongst the 59 surveyed villages, one of which was the remote Tai Dam village of Om Phalong in Mai District, Phongsaly Province in the north of the country. Abnormally high antibody prevalences of *T. solium* taeniasis (46.67%, 95% CI?=?22-72) and cysticercosis (66.67%, 95% CI?=?38-86) were detected on the initial EITB survey in this village, compared to an overall prevalence of 2.9% (taeniasis) and 4.7% (cysticercosis) in the wider survey (unpublished data). The large confidence intervals in this case reflect the relatively small sample population (n?=?16) determined under the proportional to size sampling frame for the wider provincial-level study. The requirement for follow up work in Om Phalong village to confirm the suspected hotspot was acknowledged by researchers, who returned in January 2013 to investigate further; the results of which are discussed below.

### Methods

The village of Om Phalong is situated in a remote mountainous region of Mai District in Phongsaly province, close to the Vietnamese border in northern Lao PDR. Its 330 inhabitants practice subsistence and swidden agriculture, with the village largely inaccessible for five to six months of the year during the wet season. All village members are from the Tai Dam or “Black Tai” ethnic group, an animistic culture of which the ritual slaughtering and subsequent raw consumption of pigs and buffalo occurs throughout the year around major ancestral ceremonies [10].

Sample collection took part over a two week period during the 2013 dry season, when village access was maximised and the rice harvest complete. All village members over 10 years old were invited to participate in the study, via the completion of a questionnaire (accompanied by the parent or guardian in the case of the volunteer being less than 16 years of age) and the submission of a faecal sample. Originally, serum samples were also requested to gauge the level of active cysticercosis in the village, however overlying cultural sensitivities of the Tai Dam around withdrawal of blood from the body meant only thirteen venous blood samples were collected during this time. After informed consent through a signature or thumb print, participants were given a labelled plastic bag for a faecal sample to be returned within 24 hours. Participants were identified so they could be traced back for treatment if found to be positive. Ethical approval was obtained from the Lao PDR Ministry of Health National Ethics Committee for Health Research (NECHR) [approval number 013/NECHR].

At the conclusion of every day, collected faecal samples were divided into three 10ml falcon tubes containing 10% formalin, 5% formal saline and 70% ethyl alcohol respectively, allowing for analysis by microscopy, copro-antigen ELISA and PCR. Upon return to Vientiane, samples in 5% formal saline were processed in-country by a copro-antigen ELISA (ITM, Belgium) to determine excretory-secretory proteins from adult *Taenia* species, as discussed in Allan and Craig [11]. In addition to identifying *Taenia* sp. by copro-antigen ELISA, all 92 faecal samples underwent microscopy analysis for *Taenia* sp. ova and co-existing Soil Transmitted Helminths (STH), expanding the parasitological baseline in order to demonstrate any added value to a future *T. solium* control intervention. All copro-antigen ELISA and microscopy positive samples were subsequently submitted for molecular characterisation at Murdoch University in Western Australia. Additionally, the thirteen collected blood samples were tested for human cysticercosis using the Advanced Practical Diagnostics Cysticercosis Antigen (Ag) ELISA (apDia, Belgium, Reference 650501), reporting a diagnostic sensitivity of 100 percent provided three or more viable cysts are present [12].

### Results and discussion

A total of 92 participants, representing 27.9% of the total village population, were included in the final dataset, having supplied a faecal sample, participated in the questionnaire, and complied with ethical guidelines of participation. Initial microscopy analysis identified *Taenia* spp. ova in 8 samples. All 92 samples then underwent analysis by copro-antigen ELISA, with the 8 microscopy positive samples and a further 16 samples (24 in total) testing positive, indicating a human taeniasis prevalence of 26.1% (95% CI?=?18.2-35.9). The 24 copro-antigen positive samples were then submitted for PCR at the 12S rRNA locus as per Traschel *et al*. [13] at Murdoch University in Perth, Western Australia. A generic taeniid PCR was used due to a need to detect *Taenia* sp. from more than one host (not reported here). However, only those eight samples for which *Taenia* ova were identified on microscopy amplified successfully, due to the requirement for *Taenia* DNA to be present in order for amplification to occur [14]. Of the eight samples positive by PCR five sequenced successfully, with three failing the sequencing process. All five however, identified 100% with published genetic sequences for *T. solium* [NCBI Accession numbers AB086256 and L49444]*.*

Out of the thirteen submitted blood samples, four were found to be positive, indicating a presence of circulating antigens to *T. solium* cysticerci of 30% (95% CI 9-61). Despite the large confidence interval and potential sampling bias in these thirteen samples, subsequent social research revealed that of the four cysticercosis positive patients, only one wanted to have their blood taken due to feeling “ill”, with the other three submitting blood samples because they “wanted to participate in the village activity” (Key Informant Interviews, 2013). A broader investigation under the aforementioned anthropological study revealed complaints of headaches, dizziness and subcutaneous nodules to be relatively widespread in the village (unpublished data). Despite very few reports of seizures or epilepsy from both quantitative and qualitative interview techniques, further longitudinal investigation is required, given the potentially sensitive subject matter for many ethnic groups in the region [15].

The fact that *T. saginata* and/or *T. asiatica* were not detected in any of the genotyped samples, coupled with the abnormally high EITB results on the initial 2011 survey and positive human serum-antigen ELISAs, leads us to believe that *T. solium* is indeed the predominant tapeworm within the copro-antigen positive samples. Moreover, the range of complementary diagnostic tests used in this study strongly suggests *T. solium* exists within the human population of Om Phalong at significantly higher levels than indicated in previously published studies from Lao PDR [6,7]. We consider this discovery may be the tip of the iceberg of potential hyper-endemic hotspots of *T. solium* in the country, particularly for certain ethnic groups such as the Tai Dam, for which raw pork is the preferred method of consumption during sacrificial slaughter. In terms of control in this particular village, a combined human and porcine biomedical intervention is currently underway in an attempt to significantly lower the current taeniasis burden, thus impacting on further incidence of human cysticercosis infections in the short term. In order to address the underlying sanitation issues and develop robust strategies for longer term control, social research methodologies will continue to be applied in parallel to the epidemiological investigation, in order to explore the various socioeconomic and sociocultural drivers of *T. solium* transmission in this particular ethnic group.

### Conclusion

It is envisaged that the information gained through the use of combination diagnostic and methodological approaches implemented here will assist the development of longer term, locally acceptable strategies for control, which could eventually be rolled out into other similarly affected communities in the region. Given the suspected high clustering of *T. solium* infection in Lao PDR, possibly attributed to cultural norms of raw pork eating by specific ethnic groups, the authors recommend further epidemiological and socio-cultural research approaches to refine the combination methodologies applied in this investigative study.

## Abbreviations

AAHL: Australian Animal Health Laboratory of the CSIRO; ACIAR: Australian Centre for International Agricultural Research; CSIRO: Commonwealth Scientific and Industrial Research Organisation; EITB: Enzyme-linked Immunoelectrotransfer Blot strip; ELISA: Enzyme-linked immunosorbent assay; ILRI: International Livestock Research Institute; Lao PDR: Lao People’s Democratic Republic; NTD: Neglected tropical disease; NZD: Neglected Zoonotic Disease; PCR: Polymerase chain reaction; WHA: World Health Assembly; WHO: World Health Organisation.

## Competing interests

The authors declare that they have no competing interests.

## Authors' contributions

AO, AA, BK and JA designed the study. AO, AA, CK, BK and EH undertook sample collection. AA, CK and LT conducted the laboratory analysis and AO drafted the manuscript, with subsequent input from AA, LT, EH, PD and JA. All authors read and approved the final manuscript.
